# Realization of large energy proportion in the central lobe by coherent beam combination based on conformal projection system

**DOI:** 10.1038/s41598-017-02118-z

**Published:** 2017-05-19

**Authors:** Dong Zhi, Zhixin Zhang, Yanxing Ma, Xiaolin Wang, Zilun Chen, Wuming Wu, Pu Zhou, Lei Si

**Affiliations:** College of Optoelectronic Science and Engineering, National University of Defense Technology, Changsha, 410073 China

## Abstract

In this paper, we experimentally validate a tiled-aperture conformal projection system with the largest array filling factor and element beam truncation factor to the best of our knowledge. The conformal projection system, which is made up of a hexagonal adaptive fiber-optics collimator (AFOC) array with the proximate ideal intensity distributions, is fabricated and the performance of output beam is tested and evaluated properly and carefully. Both of the active phase-locking control and precise tip-tilt control are implemented successfully in the CBC of the hexagonal seven-beam-array. Experimental results show a large energy proportion (47%, which increases by over 10% comparing with the previously demonstrated largest value) in the central lobe is achieved and the residual phase error is lower than *λ*/27. When the AFOC array performs, the precise tilt control makes the combining beams overlap well and the average normalized metric value is improved from 0.336 without control to 0.947 with both of active piston and tip-tilt phase controls while the fringe contrast increases from 19% to more than 91% correspondingly. This work presents a promising structure for the achievement of large energy proportion in the central lobe in high power fiber laser CBC systems.

## Introduction

There has been an ongoing effort to achieve a high power fiber laser with high brightness via coherent beam combination (CBC) technique^1, 2^. CBC of fiber laser array, which is a promising approach to overcome the power scaling limitations of the single fiber laser and to achieve high-brightness laser with good beam quality, can be used in beam projection area and free space laser communication system with enormous potential. CBC systems mainly contain two subsets, which are characterized by the output formatting, tiled-aperture and filled-aperture implementations^1–3^. Comparing with filled-aperture format, CBC based on tiled-aperture-stitching method has the advantage free of power limitation induced by single optical component (diffraction optical element, self-imaging waveguide, polarization beam combiner, *et al*.) and the disadvantage of energy wastage in the side-lobes in the far-field pattern^4–7^. The side-lobes are the direct results of lower energy proportion in the central lobe, which is an important character concerned in most CBC applications. For the tiled-aperture combination scheme, the energy proportion of side-lobes is determined by the assembling of the beam array, which is difficult to select and to implement^8, 9^. This assembly requires a trade-off between an under-filled aperture which suffers from significant side-lobes in the far-field, and an over-filled aperture which suffers from clipping power loss in the near field^9^. Lots of tiled-aperture-based CBC experiments focusing on the active phasing algorithms have been carried out and demonstrated well with high fringe contrast and control accuracy^8–13^. However, due to the less-than-unity filling factor of beam array and the small truncation factor of single beam element, the energy proportion in the central lobe has been small^11–15^. Up to now, the largest energy proportion in the central lobe experimentally achieved is almost 60%, which is coincidence with the theoretical calculation value, by J. Anderegg in 2006 based on tiled-aperture CBC architecture utilizing bulk free space optical elements^16, 17^. In order to decrease the complexity, researchers use the integrative fiber collimator to carry out CBC experiments^18–22^. At the same time, the filling factor of the integrated collimator array is usually below 70% and the main-lobe energy proportion is usually below 30%^19–22^. To improve the array filling factor, researchers utilize the microlens array to arrange the array beams^23–26^. Using this method, J. Bourderionnet and his co-workers improved the energy proportion in the main lobe to be 34% for two-dimensional beam array and C. X. Yu *et al*. demonstrated a CBC of one-dimensional beam array with 58% central-lobe energy proportion, which means the value should be 58%^2^ = 33.6% for two-dimensional beam array situation^25, 26^. Here the considerable power fraction in the side-lobes mainly comes from the Gaussian beamlet wings, namely small truncation factor of each element. In general, the appearance of side-lobes mainly comes from the less-than-unity intensity distributions in the near field of the laser beam array, which is difficult to precisely design and measure.

In all of practical CBC systems, tilt phase errors of the array beams is another important factor that influences the energy proportion in the central lobe, but has been seldomly considered especially in high power situation^8, 27–30^. Tilt phase errors, which are mainly induced by atmospheric turbulence and vibration of mechanism, must be taken into consideration even if the active piston phasing performs well^8, 27, 28^. Adaptive fiber-optics collimator (AFOC) is an effective way to compensate the small tip-tilt aberrations with high precision and achieves rapid development in recent years^27–33^. Conformal projection system based on AFOC array has not only the excellence to compensate tilt errors of beamlets but also many other advantages over telescope projection system with a big monolithic mirror^34^. Our group has previously demonstrated the control performance and high-power-support capability of homemade AFOC based on flexible hinges and levers, which control the fiber end cap instead of the bare fiber^35^.

In this manuscript, a hexagonal conformal projection system with the largest array filling factor and the largest single beam truncation factor to the best of our knowledge is presented and demonstrated. In the CBC experiment, a large energy proportion (47%, which increases by more than 10% comparing with the largest value using lens-array combining method) in the central lobe is achieved and the residual phase error is lower than λ/27. Moreover, simultaneously active phase-locking control and precise tip-tilt control are demonstrated successfully based on the hexagonal tiled-aperture CBC system. When the AFOC array performs, the precise tilt control makes the combining beams overlap well and more concentrated in the far-field.

## Results

### Design and performances of the conformal projection system

**Figure 1:**
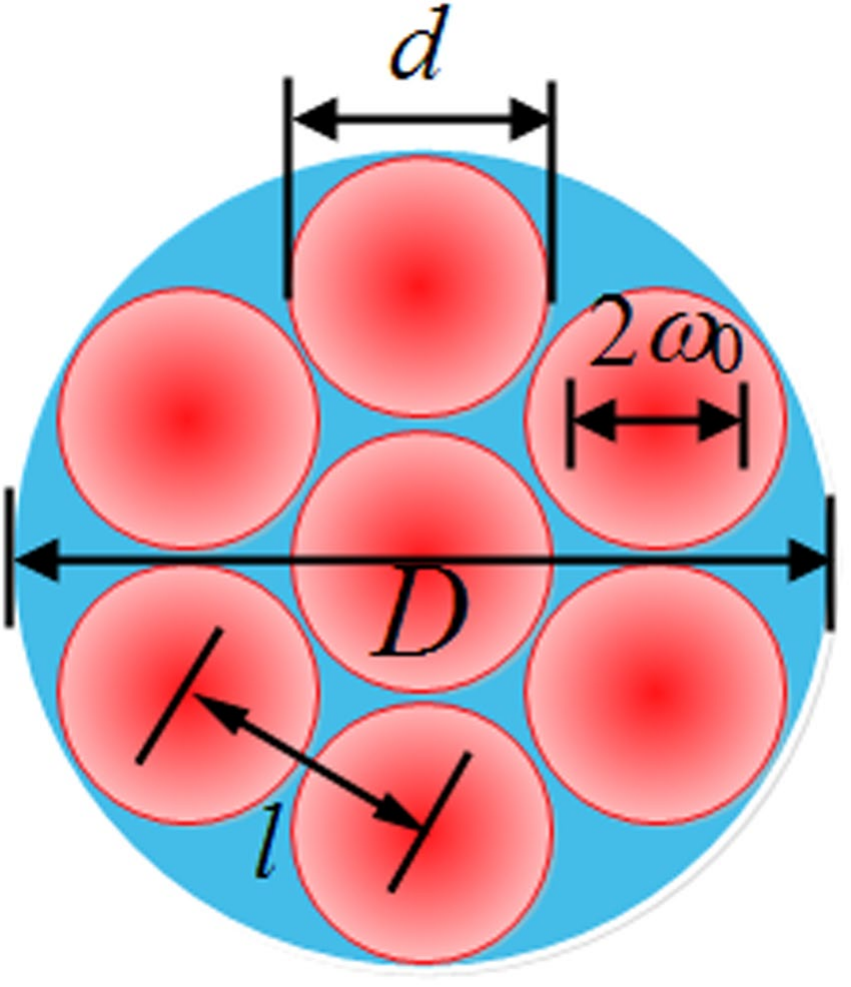
The diagram of a tiled combining beam array.

In the design work, the most important factor to improve the energy proportion in the central lobe is the array beams near-field filling factor, which can be divided into sub-aperture filling factor and conformal filling factor^8, 36^. The essential goal is close to get the plane-wave-like near-field intensity distribution. For definiteness, we consider our conformal projection system in the form of a hexagonal array of lens-based fiber collimators with circular apertures (sub-apertures) with diameter *d* and the distance between the centers of adjacent sub-apertures *l*, just as depicted in Fig. 1. Then the conformal filling factor can be calculated as *d*/*l*. The clear aperture of the hexagonal array is *D*, which is equal to 2*l* + *d*. As the beam waist of each collimated beam is *ω*_0_, the sub-aperture filling factor is 2*ω*_0_/*d*, which is also known as truncation factor. As the structure dimension of AFOC used here is limited to 50 mm × 50 mm, we design *l* to be 60 mm^33^. The conformal filling factor is the first parameter to concern about, the larger the better. So we design the thickness of the collimator pipe to be 1 mm and the collimator aperture is 58 mm with the conformal filling factor of 96.7%, which is the largest array filling factor in the public literatures.

**Figure 2:**
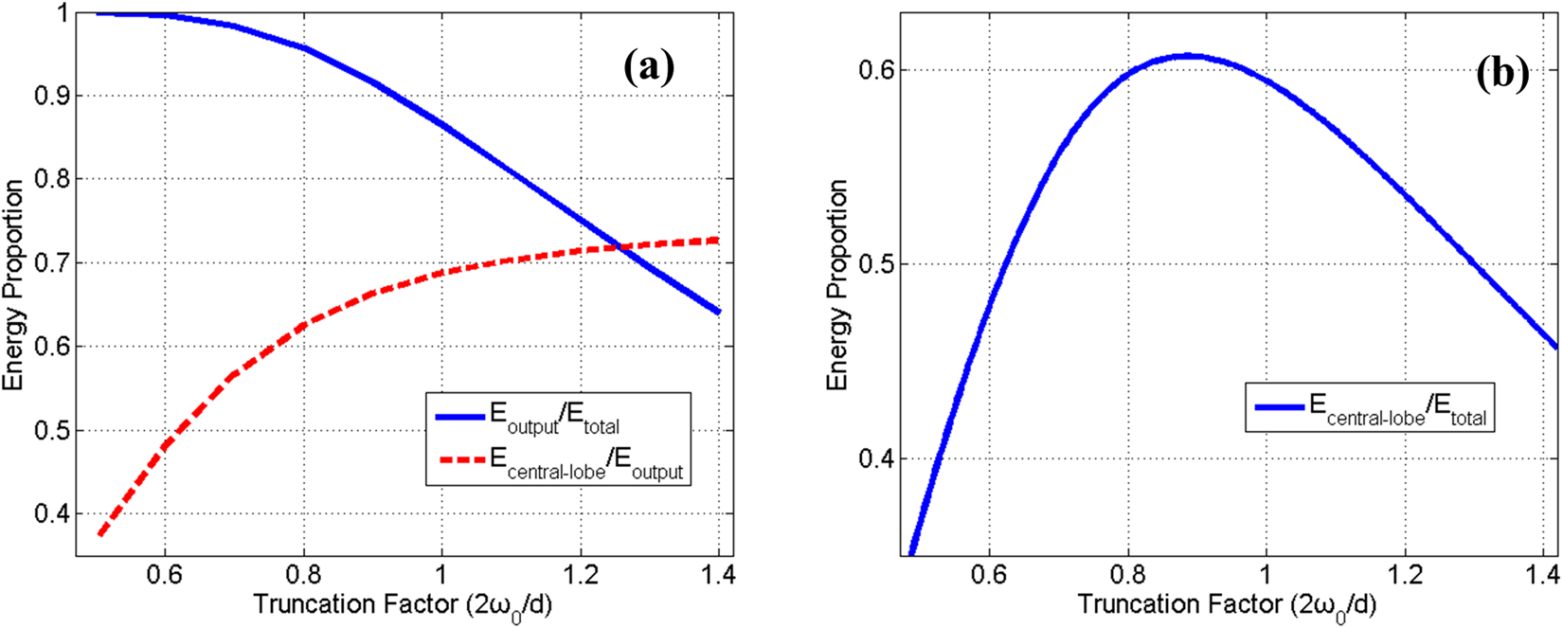
The energy proportion as the function of truncation factor.

The sub-aperture filling factor should be calculated and measured carefully. As we mentioned in ref. [35], when the focal length *f* is 180 mm, the beam waist of collimated beam is about 7.3 mm to the situation of 20/400 output fiber with core numerical aperture (NA) of 0.065. So the beam size (2*ω*_0_) to *f*·NA ratio is 1:1.24. Using the same measurement method, we have measured the ratio to be about 1:1.1 with 25/250 output fiber. The value of focal length directly influence the truncation factor when the clear aperture is fixed. The truncation factor affects power ratio of the output laser and intensity distributions of the laser array. Just as depicted in Fig. 2(a), the larger truncation factor, the less output energy proportion (E_output_/E_total_) and the closer distribution to unity, which caused the larger ratio of central-lobe energy to output energy (E_central-lobe_/E_output_). The relationship between the ratio of central-lobe energy to the total energy (E_central-lobe_/E_total_) and the truncation factor is shown in Fig. 2(b). Energy proportion in the central lobe has a maximal value of about 0.62 when the truncation factor is about 0.9. Here we choose the focal length of collimating lens *f* to be 800 mm, and the corresponding truncation factor 0.91 is close to the optimal value.

**Figure 3:**
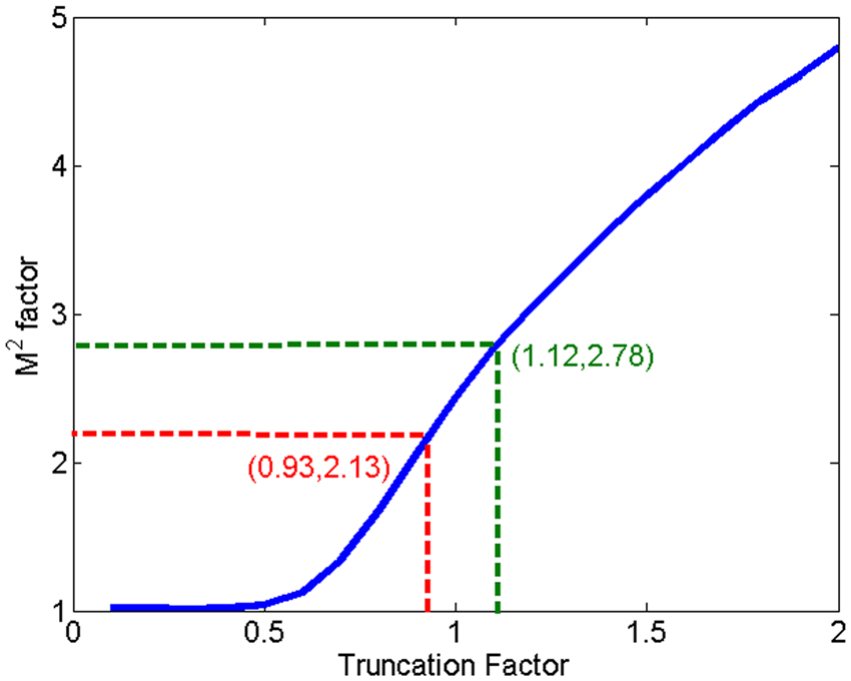
The classic M2 factor of the truncated Gaussian beam as the function of truncation factor.

After we aligned the lens and the fiber end-cap positioner accurately, we tested the abilities of the collimated beam. By carefully measuring and calculating the near-field and far-filed intensity distributions in the same measurement method in ref. [35], we obtained the beam waist width of the collimated beam *ω*_0_ = 27 mm with corresponding truncation factor of 0.93. The far-field beam waist width *ω*_*far*_ is measured to be about 625 μm by using a focusing lens with focal length *f*_1_ of 20 m. Then the beam quality factor (M^2^ factor) is calculated to be π·(*ω*_0_·*ω*_*far*_)/(*λ*·*f*_1_) = 2.49. The reason that the measured M^2^ factor is much larger than the ideal Gaussian beam is mainly because the beam is truncated by a hard edge aperture. We numerically calculate the M^2^ factor of truncated Gaussian beam as the function of truncation factor, which is shown as the blue line in Fig. 3. To evaluate the beam quality of truncated Gaussian beam properly and conveniently, we introduce a modified M^2^ factor, which is defined as the ratio of measured value to the theoretically calculated value. The basic principle of this modified M^2^ factor is the comparison of the product of far-field divergence angle and beam width between the actual and the ideal situations, which is similar to the definition of classic M^2^ factor. The modified M^2^ factor is always larger than unity, and the smaller value the better beam quality. Using this definition, the modified M^2^ factor is 2.49/2.13 = 1.17, which represents a good beam quality.

**Figure 4:**
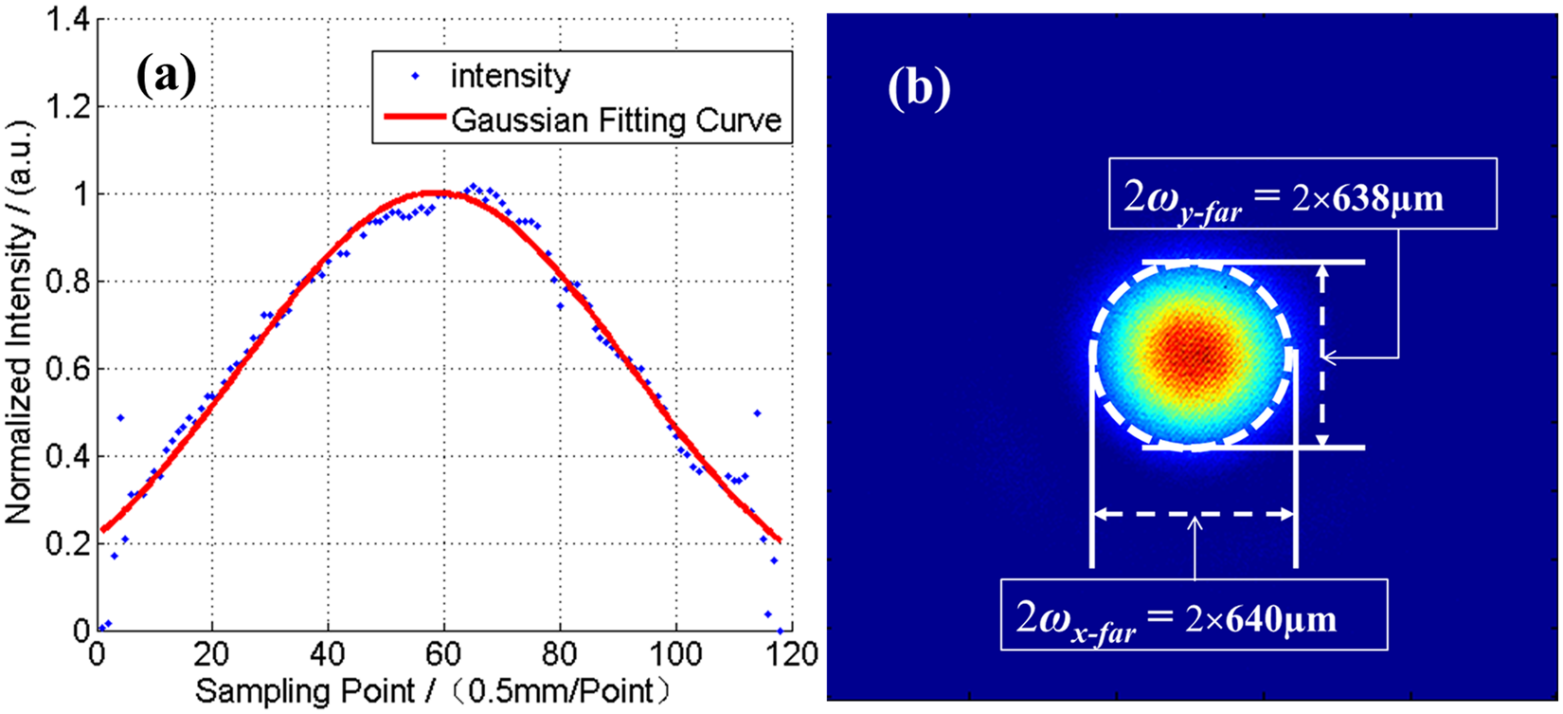
The intensity distributions of the near-field (**a**) and far-field (**b**) of the collimated beam with 20/400 output fiber.

To the quantitative limitation of fiber amplifiers, we use a fiber mode field adaptor (MFA) to connect the single mode fiber and the multimode fiber with large mode field area. As the mismatching of core NA in MFA and the 25/250 fiber supports many eigen modes, the laser beam will inevitably excite a considerable part of high order modes when transmitting the MFA, which is not desirable in CBC of beam array. So in the following CBC experiments, we choose the output fiber to be 20/400 fiber with core NA of 0.065, which only supports LP01 and LP11 eigen modes. When using 20/400 fiber to optimize the output beam quality and the unchangeable collimating lens with focal length of 800 mm, we measure the beam waist width of the near-field and far-field of the collimated beam to be 32.5 mm and 640 μm with corresponding truncation factor of 1.12, just as shown in Fig. 4. Then the classic and modified M^2^ factors are calculated to be 3.07 and 1.10 correspondingly. Here the truncation factor of 1.12 is the largest value reported public to the best of our knowledge.

Then we test the performances of AFOC. The deflection angles of the collimated beam emitted from the AFOC are about in range of 0~0.05 mrad (40 μm/*f* = 0.05 mrad) for the chosen focal length *f* = 800 mm. The first resonance frequency of the AFOC is about 700 Hz.

### Experimental setup of CBC

**Figure 5:**
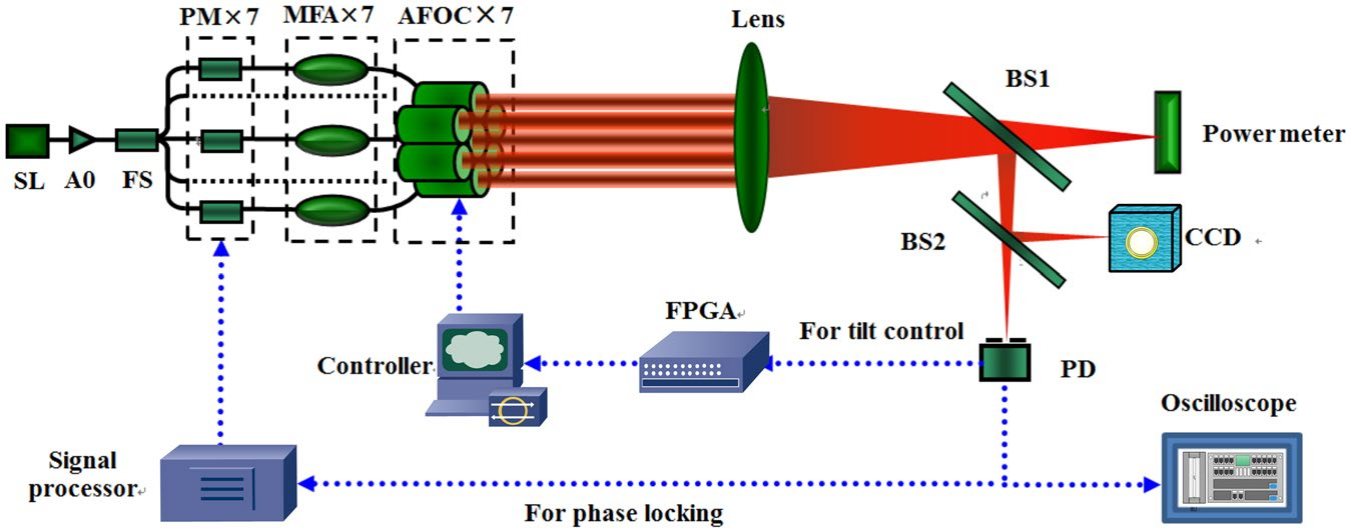
Experimental setup (SL: seed laser, FS: fiber splitter, PM: phase modulator, MFA: mode field adaptor, AFOC: adaptive fiber-optics collimator, BS1: 90:10 beam splitter, BS2: 50:50 beam splitter, PD: photonic detector).

A master-oscillator-power-amplifier-based CBC experiment platform as shown in Fig. 5 is setup to validate the performance of the active piston and tip-tilt control based on our homemade hexagonal AFOC array. The seed laser (SL) is a Yb-doped fiber laser at 1064.55 nm wavelength with output power above 30 mW and line width below 20 MHz. The SL is pre-amplified by A0 to above 400 mW. Then the pre-amplifier laser is split into 8 channels by fiber splitter (FS) with output power of 50 mW per channel. Seven channels were chosen for CBC experiment and each of them connects a LiNbO_3_-based phase modulator (PM). After that a mode field adaptor (MFA, the input fiber is 10/125 and output fiber is 20/400) is used in each channel to connect the PM with AFOC, which acts as the output terminal of fiber laser and utilizes fiber end-cap with 20/400 fiber. A lens with clear aperture of 200 mm and focal length of 20 m is used to focus the seven laser beams in the far field. The combined laser beam is split into three parts by a 90:10 beam splitter (BS1) and a 50:50 beam splitter (BS2). The 90% portion of the combined beam is collected by a power meter. The second part of the combined beam is imaged on a CCD camera to observe the far-field combined beam patterns. The third part of the passing light is detected by a photonic detector (PD) with 150 MHz bandwidth to provide feedback data. A pinhole with diameter of 80 μm is located in front of the PD.

Throughout the experiment, the metric function is the voltage provided by the high-speed PD, which represents the far-field beam intensity in the pinhole, or called power in a restricted bucket (PIRB). The feedback signal is sent to a signal processor for phase-locking control and a field programmable gate array (FPGA) processor for precise tip-tilt control simultaneously. The iteration frequencies for active phasing control and tip-tilt control are 100 MHz and 400 Hz, respectively. Stochastic parallel gradient descent (SPGD) algorithm [12] and single frequency dithering algorithm [13] are used to perform the tip-tilt control and the phase-locking control individually.

### Validation of active phase-locking control

To validate the performance of active phasing control independence of tip-tilt errors, we manually eliminate the initial tilt phase aberrations among these seven combining beams. Experimentally, we adjust each centroid of the array beams to overlap at one point in the far-field. The time series signal recorded by the PD contains the information of phase differences among the seven fiber laser chains, which can be used to generate the phase control signal to the PM and to evaluate the performance of phase-locking controller.

**Figure 6:**
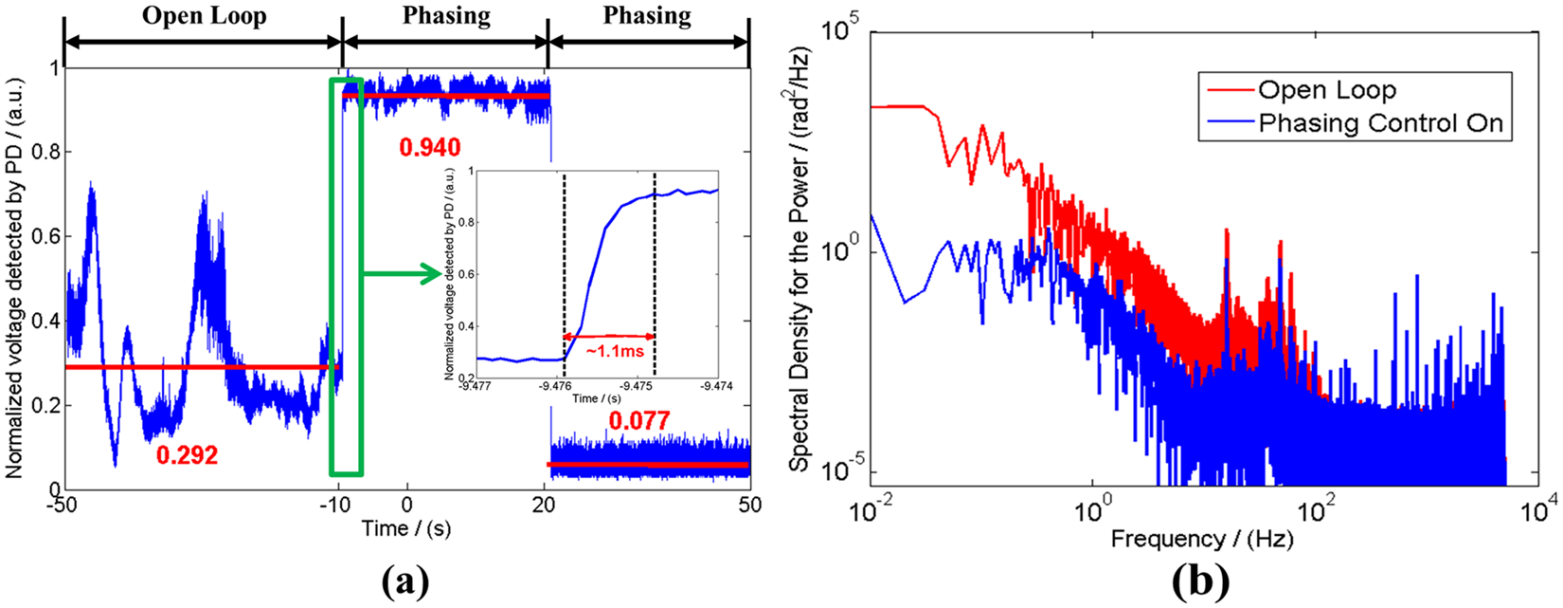
The normalized voltage values detected by the PD along with time and the spectral density of power of phase noise with different situations. (**a**) Time-dependent phasing process. **(b**) Spectral density.

Figure 6(a) shows the normalized voltage values detected by the PD in different situations, which represents the time domain variation of metric function PIRB. The metric function fluctuates randomly along with time in open loop (without active phasing control), which is ascribed to that phase differences among the seven laser channels change with time. Its mean value is about 0.292 in nearly 40 seconds. When choosing the maximum metric value as the algorithm optimized direction and starting the phase-locking control, the normalized value of metric function climbs steeply up to above 0.9 in about 1.1 milliseconds. By statistical analysis, we derive the mean value is about 0.940 in almost 30 seconds and the root mean square (RMS) value is 0.014, which can be used to calculate the residual phase errors to be about λ/27. When the phasing-locking controller performs at the minimum optimized direction, the mean value of the PIRB and RMS are 0.077 and 0.012, respectively. From the detected time domain signal of the two phasing states, we can verify the phase differences among the seven lasers are compensated effectively and the phase-locking processor works well with high accuracy. Figure 6(b) shows the spectral density of power of phase noise both in open loop and in phasing state. From the comparisons of the two situations, we can conclude that the phase noise with characteristic frequency below 100 Hz can be compensated efficiently when the phasing control is on.

**Figure 7:**
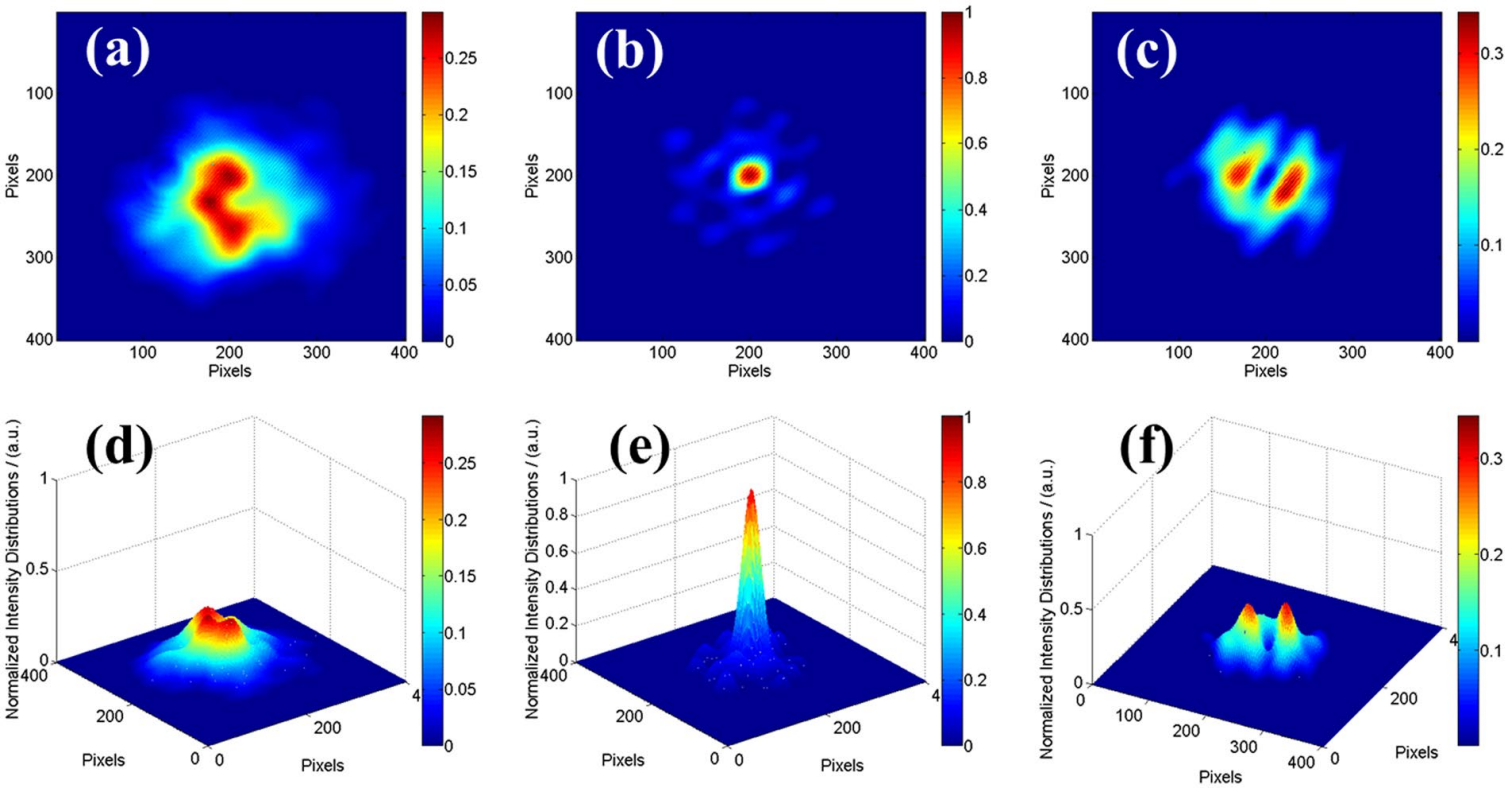
The long exposure beam pattern in different situations. (**a**)~(**c**) Two dimensional intensity distributions. (**d**)~(**f**) Three dimensional intensity distributions.

The performance of the phase-locking controller can also be directly tested and verified by the long exposure beam pattern from the CCD camera. Figure 7(a)~(c) (Fig. 7(d)~(f)) shows the normalized two dimensional (three dimensional) beam intensity distributions in the far-field for 30 seconds long exposure of different states corresponding to Fig. 6(a). Fig. 7(a) and (d) present the far-field beam patterns in the open loop with wide distribution area, weak brightness and low contrast, which is similar to the incoherent combining effect. As depicted in Fig. 7(b) and (e), the coherent pattern is obviously stable with high fringe contrast above 98% and large energy portion in the central lobe. By calculation we obtain a high brightness coherent laser beam with more than 47% energy portion in the central lobe. Comparing with the ideal energy portion of 70% in the central lobe with truncation factor of 1.12 shown in Fig. 2(a), the experimental efficiency is above 67%. The loss of efficiency mainly comes from the high order mode induced by the MFA in each channel and the high order aberrations, like comatic aberration, introduced by the focusing lens with finite aperture and the non-paraxial peripheral six array beams. When the phasing control works at the minimum state, there is an interesting intensity distribution with a dark hollow shown in Fig. 7(c) and (f). As the phase differences of the combined beams finally are controlled in a state that satisfies the detected point is lower in intensity, this hollow-dark-like beam is stable in time domain. So this hollow beam can be used in some applications like high resolution imaging, biomedical engineering, special material processing and so forth.

### Performance of precise tip-tilt control

Apart from the piston phase errors compensation, which is none the less the most important consideration in lots of CBC systems, tip-tilt phase aberrations controlling also plays an important role in most actual applications, even if the piston phase is locked effectively. In practical systems, tip-tilt phase aberrations mainly come from the limited assembling precision of AFOC array, vibration of mechanism, atmospheric turbulence effects, *et al*. To investigate the importance of precise tip-tilt phase errors compensation and to validate the performance of the AFOC array, we introduce small tip-tilt phase aberrations over each of the seven beams and start over both of the piston and tip-tilt phase control systems.

**Figure 8:**
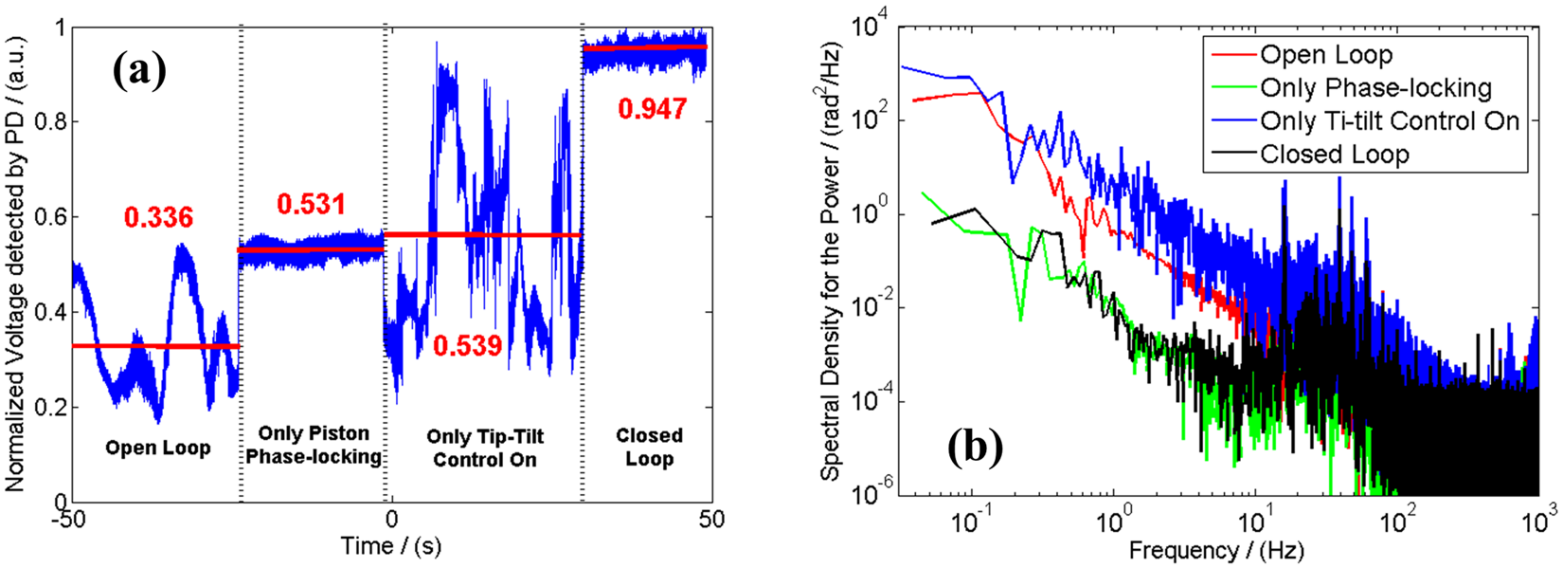
The normalized voltage and spectral density of power of phase noise with/without active phasing control and/or tip-tilt control. (**a**) Time domain normalized voltage signal. (**b**) Spectral density.

The normalized voltage recorded by the PD as the function of time in different situations is shown in Fig. 8(a). The metric function in open loop (without tip/tilt control and phase-locking control) fluctuates within a small range with mean value of 0.336. When the phase-locking control is performing, the time-dependence signal has remained stable with a higher average value of 0.531. After that we start the tip/tilt control and close the phase-locking control, the detected signal with the normalized average value of 0.539 fluctuates heavily along with time, which represents the amplification of phase differences turbulence among the seven laser chains. In closed loop (both of the phase-locking control and the tip/tilt control are performing), the average of the normalized PIRB increases to 0.947 and the RMS decreases to 0.012 with the residual phase errors lower than *λ*/28, which means that the two controls have been achieved successfully and the homemade AFOC array works well. Figure 8(b) shows the spectral density of power of phase noise in different situations corresponding to Fig. 8(a). From Fig. 8(b), we can conclude that the tip-tilt control can improve the spectral density of phase noise, while phase-locking control can suppress the spectral density.

**Figure 9:**
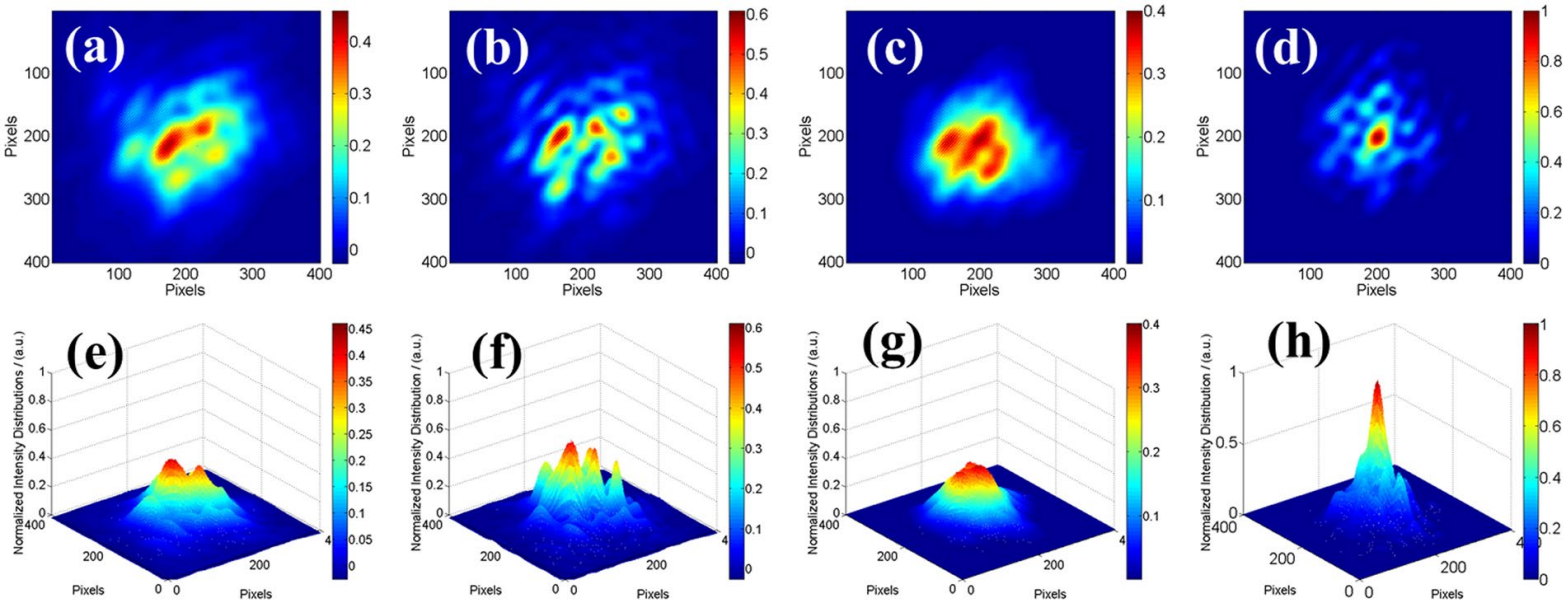
Long exposure beam intensity distribution patterns in different situations. Top row: Two dimensional intensity distributions. Bottom row: Three dimensional intensity distributions. (**a** and **e**) Open loop, (**b** and **f**) Only the phase-locking control is on, (**c** and **g**) Only the end-cap/tilt control is on, **(d** and **h**) Closed loop).

To directly observe the feasibility of the two controls, we derive the long exposure (30 seconds) far-field combining beam pattern for different stages as shown in Fig. 9 corresponding to Fig. 8(a). Figure 9(a)/(e) and (b)/(f) present the far-field beam patterns without and with the phase-locking control, respectively, but both without tip-tilt control. As depicted in Fig. 9(a) and (b), the combined beam distributes in a large area in the far-field, even though the fringe contrast increases from 19% to 66% due to the phase-locking control. As the tip-tilt errors are small, the seven combining beams are coherent with each other and the interference fringes are much complicated. As the seven beams are unvisibly separated in the far-field, the precise tip-tilt control cannot be realized manually. When the tip-tilt controller is performing, the seven beams are more concentrated in the far-field just as depicted in Fig. 9(c)/(g). Furthermore, when the two controls are both on, the long exposure far-field intensity distributions is shown in Fig. 9(d)/(h). It reveals that the fringe contrast increases from about 36% (Fig. 9(c)) with only the tilt control to more than 91% (Fig. 9(d)) with both two active controls on. For Fig. 9(d), energy portion in the bucket with an area equal to Airy disk is calculated to be 35%, in contrast to 15% in the case of Fig. 9(b) which also incorporates active piston phasing control. This means that a higher brightness laser beam is achieved. The reduction of the energy portion in the central lobe of the situation shown in Fig. 9(d) compared with Fig. 7(b) is mainly due to the residual tip-tilt errors and control precision of the optimized algorithm and devices.

By the analysis above, we see that the precise tip-tilt control can make the combining beams overlap well within a tiny residual tilt aberrations. The precise tip-tilt control is important for extracting the phase differences fluctuations of the combining beam array and has great influence on the CBC effect, hence should be taken into consideration especially in high power practical CBC systems.

## Discussion

In conclusion, we fabricate a hexagonal AFOC array with the largest array filling factor and the largest element-beam truncation factor. To evaluate the beam quality of truncated Gaussian beam properly and conveniently, we introduce a modified M^2^ factor. Utilizing this new metric to evaluate the output beam, we measure the modified M^2^ factor to be about 1.1 representing good beam quality. Then CBC of hexagonal seven-beam system is achieved with a large energy proportion (47%) in the central lobe and a residual phase error lower than λ/27 in the experiment. Moreover, when the phasing control works at the minimum state, we obtain a dark-hollow-like beam. Furthermore, the CBC of seven beams with simultaneous active phase-locking control and precise tip-tilt control is demonstrated. The AFOC array is employed here to implement the tilt errors compensation for the hexagonal tiled-aperture CBC system. Experimental results show that the precise tilt control makes the combining beams more concentrated and overlap together. The average normalized PIRB value is improved from 0.336 in open loop to 0.947 in closed loop while the fringe contrast increases from 19% to more than 91% correspondingly. The hexagonal AFOC array with a large truncation factor is a promising way to generate a large energy proportion for many applications using high power fiber laser, such as free-space communications and beam projection systems, *et al*.

In the future, when the densely packed AFOC array is working under high output power situation (kW level), the treatment of the considerable amount of waste heat is very important and crucial. The waste heat mainly comes from the truncated beam tail, which plays a direct role in heating the collimator tube. Moreover, in order to reduce the thermal lens effect of optical elements, cooling of the fiber end-cap and collimating lens also should be taken into consideration for high power systems.

## Method

### Measurement Method

A CCD camera with pixel size of 4.4 μm × 4.4 μm from Spiricon Inc. is used as a two-dimensional detector array for the patterns detection of the output combined beam. A gain adjusting PD with the rise time of 25 ns and bandwidth of 150 MHz is employed to provide the feedback signal to the algorithms controllers. Temporal domain characteristics of different situations are detected by employing a 0.5 G Hz oscilloscope.
